# Local and systemic gene expression responses of Atlantic salmon (*Salmo salar L*.) to infection with the salmon louse (*Lepeophtheirus salmonis*)

**DOI:** 10.1186/1471-2164-9-498

**Published:** 2008-10-23

**Authors:** Stanko Skugor, Kevin Alan Glover, Frank Nilsen, Aleksei Krasnov

**Affiliations:** 1 Nofima Akvaforsk Fiskeriforskning, P.O.Box 5010, Ås 1430, Norway; 2 Institute of Marine Research, PO Box 1870, Nordnes, 5817 Bergen, Norway; 3 Department of Biology, University of Bergen, Box 7800 N-5020 Bergen, Norway

## Abstract

**Background:**

The salmon louse (SL) is an ectoparasitic caligid crustacean infecting salmonid fishes in the marine environment. SL represents one of the major challenges for farming of salmonids, and veterinary intervention is necessary to combat infection. This study addressed gene expression responses of Atlantic salmon infected with SL, which may account for its high susceptibility.

**Results:**

The effects of SL infection on gene expression in Atlantic salmon were studied throughout the infection period from copepodids at 3 days post infection (dpi) to adult lice (33 dpi). Gene expression was analyzed at three developmental stages in damaged and intact skin, spleen, head kidney and liver, using real-time qPCR and a salmonid cDNA microarray (SFA2). Rapid detection of parasites was indicated by the up-regulation of immunoglobulins in the spleen and head kidney and IL-1 receptor type 1, CD4, beta-2-microglobulin, IL-12β, CD8α and arginase 1 in the intact skin of infected fish. Most immune responses decreased at 22 dpi, however, a second activation was observed at 33 dpi. The observed pattern of gene expression in damaged skin suggested the development of inflammation with signs of Th2-like responses. Involvement of T cells in responses to SL was witnessed with up-regulation of CD4, CD8α and programmed death ligand 1. Signs of hyporesponsive immune cells were seen. Cellular stress was prevalent in damaged skin as seen by highly significant up-regulation of heat shock proteins, other chaperones and mitochondrial proteins. Induction of the major components of extracellular matrix, TGF-β and IL-10 was observed only at the adult stage of SL. Taken together with up-regulation of matrix metalloproteinases (MMP), this classifies the wounds afflicted by SL as chronic. Overall, the gene expression changes suggest a combination of chronic stress, impaired healing and immunomodulation. Steady increase of MMP expression in all tissues except liver was a remarkable feature of SL infected fish.

**Conclusion:**

SL infection in Atlantic salmon is associated with a rapid induction of mixed inflammatory responses, followed by a period of hyporesponsiveness and delayed healing of injuries. Persistent infection may lead to compromised host immunity and tissue self-destruction.

## Introduction

The salmon louse (SL), *Lepeophtheirus salmonis*, is a marine ectoparasitic caligid crustacean infecting wild and farmed salmonids of the genera *Salmo*, *Salvelinus *and *Onchorhynchus *[1]. The life cycle consists of two planktonic larval stages, an infectious stage where copepodites attach to a suitable host, 4 immobile chalimus stages where the louse is firmly attached to the host's skin or fins, then 2 free-moving pre-adult stages before the final adult stage [1,2]. Heavy infestations present one of the major problems faced in marine salmon aquaculture and the concomitant rise of epizootics in wild populations is causing much debate about the possible ecological impacts of farmed fish [3,4].

Salmon lice feed on host mucous, skin tissue and blood. Juveniles usually cause only abrasive wounds on the host skin but nevertheless lead to systemic stress responses and modulations of the immune system and physiology (reviewed in [1,5,6]). Host susceptibility differs among the salmonid species [7-10]. Coho salmon (*O. kisutch*) successfully expels parasites during chalimii stages while Atlantic salmon (*S. salar*) fails to initiate inflammation and shows no apparent tissue responses to the attached larvae [8,11]. The ability to expel parasites can be determined with hyperplastic and inflammatory responses in the epidermis and underlying dermal tissues [1] and references therein. Hyperinflammatory phenotype in the resistant coho salmon is manifested within a day post infection and is characterised by a rich neutrophil influx at the site of parasite attachment. The inflammatory infiltrate persists during the whole period of salmon lice development on coho salmon and is accompanied with the pronounced epithelial hyperplasia that in some cases completely encapsulates the parasite. Intraspecific comparisons revealed the association of an early regulation of pro-inflammatory interleukin (IL)-1β, IL-8 and tumour necrosis factor-α (TNF-α) with the improved chalimus expulsion in resistant species, which was attributed to the exaggerated T helper 1-type (Th1) responses (normally targeting viruses and bacteria) [12].

To elucidate the factors that determine high susceptibility of Atlantic salmon to lice and to evaluate the side consequences of infestation we conducted gene expression analyses throughout the life cycle, from copepodids to pre adults. Samples of damaged and intact sites of skin and immune organs (spleen, head kidney and liver) were collected 3 days post infection (dpi), 22 dpi, and 33 dpi; these time-points corresponded to the key stages (respectively copepodids, chalimus III/IV, preadult females and males). Multiple gene expression profiling is especially efficient in studies of scantily investigated conditions that may involve interactions of multiple factors. We used a high density salmonid fish cDNA microarray (SFA2 or immunochip) designed specifically for studies of responses to stressors and pathogens. In comparison with previous version ([13,14], GEO GPL1212), the updated platform was substantially enriched in immune genes (see[15]; GEO GPL6154). Real-time qPCR analyses were performed to validate the microarray results and to expand studies by examination of genes that were not present on the platform.

## Results

### Responses to salmon lice in skin

**Figure 1:**
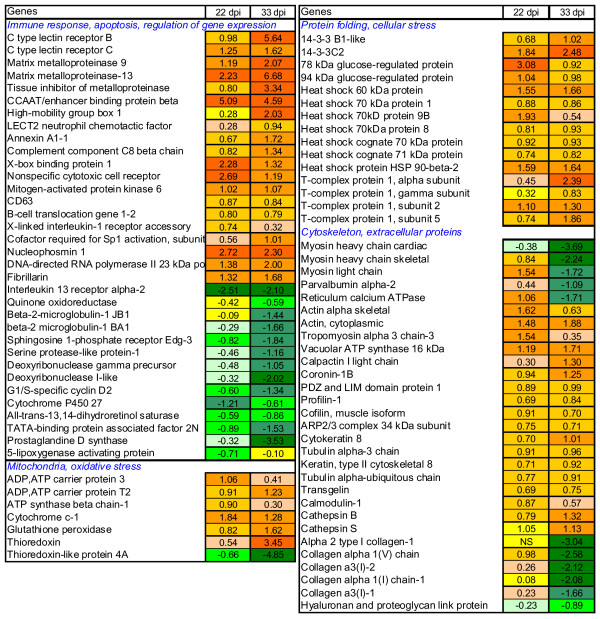
**Microarray comparison of gene expression in damaged and intact skin of SL infected fish, examples of differentially expressed genes. **Pooled samples were analysed, data are log-ER (expression ratios). Significantly up- and down-regulated genes (p < 0.01, t-test, 12 spot replicates per gene) are highlighted with red and green scales, NS means not significant.

**Figure 2:**
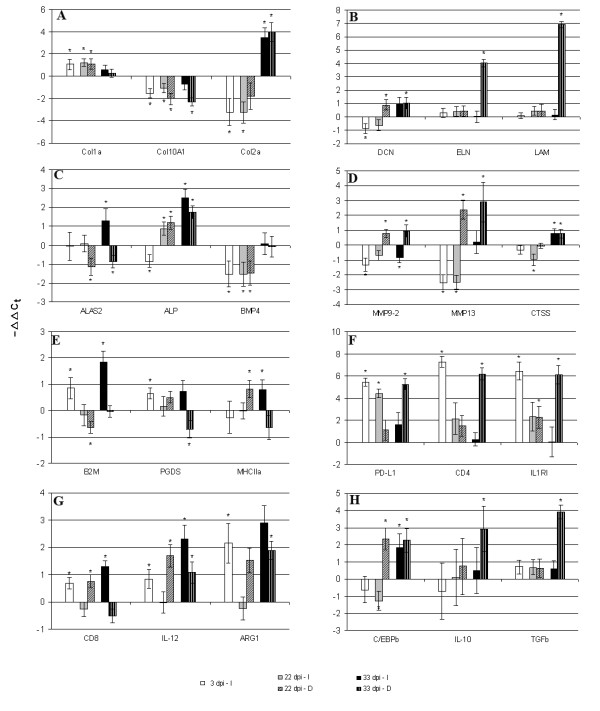
**Gene expression in skin analyzed with qPCR (individual samples).** Data are -ΔΔCt ± SD (n = 6). **A**. collagen (COL) genes: COL1a, COL2a and COL10a **B**. components of the extracellular matrix (ECM): decorin (DCN), elastin (ELN) and laminin (LMN) **C**. 5-aminolevulinate synthase (ALAS2), alkaline phosphatase (ALP) and regulatory bone morphogenic protein (BMP4) **D**. proteases involved in ECM remodelling, matrix metalloproteinases (MMP) 9 and 13 and in antigen presentation, cathepsin S (CTSS) **E**. beta-2-microglobulin-2 JB1 (B2M), a component of the major histocompatibility complex (MHC) class I; MHC class II α chain (MHCIIa) and regulator of inflammation prostaglandine D synthase (PGDS) **F**. T cell-inhibitory programmed death ligand 1 (PD-L1), CD4, marker of T cells and IL-1 receptor type 1 (IL1RI), transducer of pro-inflammatory signals **G**. CD8α, expressed on cytotoxic T cells; IL-12β, produced in Th1 settings and ARG1, marker of the Th2 response H. CCAAT/enhancer-binding protein β (C/EBPb) involved in the control of cell proliferation and differentiation; regulatory cytokines IL-10 and transforming growth factor-β (TGF-β). Significant difference from uninfected control (t-test, p < 0.05) is indicated with *. I-intact skin of infected fish, D-skin damaged by sea lice

Samples of intact skin were collected from the infected salmon within the whole study period, however, it was not possible to sample damaged skin before 22 dpi (see Materials and Methods, Fig. 7). Microarray analyses were designed to contrast the direct and indirect responses of infection with SL. Comparison of infected and intact sites from the same fish (Fig. 1) subtracted systemic responses and revealed the direct effects of parasites. Microarray comparison of intact skin from challenged and control fish was conduced to evaluate the systemic responses, however, expression changes were small (data not shown). The qPCR analyses were performed for damaged and intact skin, using skin from uninfected fish as control (Fig. 2). Both methods revealed profound changes in gene expression post infection.

Microarray analyses found differential expression of a number of genes linked to immunity. Ca-dependent (C-type) lectin receptor B and lectin receptor C, which were up-regulated in SL damaged skin, can take part in a multitude of biological processes. They regulate cellular interactions, including migration and adhesion of antigen presenting cells with lymphocytes [16,17]. In mammals, different dendritic cell (DC) subsets and maturation stages display distinct C-type-lectin profiles, depending on the local microenvironment and pathogen products [18,19]. Matrix metalloproteinases (MMP) 9 and 13 are commonly quiescent at the healthy state and become activated post-injury. These enzymes, working in conjunction with tissue inhibitors of metalloproteinases (TIMPs) (Fig. 1) can be involved in a wide range of processes such as cleavage and activation of cytokines, release of cytokines and growth factors from extracellular matrix (ECM), and establishment of a gradient for migration of cells [20]. Additionally, MMPs play the key role in the remodelling and destruction of ECM [21]. Both MMP9 and MMP13 displayed similar expression profiles, characterized by opposite regulation in the intact and damaged sites (Fig. 1 and Fig. 2D). The CCAAT/enhancer-binding protein (C/EBP) family of transcription factors are involved in positive and negative control of cell proliferation and differentiation and immune responses [22](Fig. 1 and Fig. 2H). High-mobility group box protein, a proinflammatory cytokine with DNA binding properties [23] was up-regulated 33 dpi (Fig 1).

Down-regulation of a decoy receptor IL-13 receptor alpha-2 (IL13Rα2) implied induction of the IL-4/IL-13 axis [24] and possible polarization towards the T helper 2-type (Th2) immune response, a typical response against parasites. The T helper cells (Th) that differentiate into the Th2 subset are characterised by their ability to suppress development of the IL-12 producing Th1 subset [25]. Therefore, we included in qPCR analyses the key genes that could give information on the type and dynamics of T cell responses throughout the study period (Fig. 2E, F, G, and 2H). Marked increase of CD4 transcripts 3 dpi at intact sites supports a rapid infiltration of T cells into the skin after the exposure to copepodids (Fig. 2F). High expression of CD4 at injured sites 33 dpi indicates a second wave of T cell migration from lymphoid organs or an increased proliferation in the periphery. CD4+ Th cells are essential intermediaries of the adaptive immune system, which instruct innate effector cells and amplify their responses mainly through the secretion of specific cytokines. IL-1 receptor type 1 (IL1RI) transduces signals from proinflammatory cytokines IL-1β and IL1α and can serve as a marker of a newly described highly inflammatory effector Th subset, Th17 [26]. The IL1RI expression profile was similar to that of CD4 (Fig. 2F). IL1RI was highly responsive to SL induced damage whereas its expression steadily declined in the intact sites after the initial increase 3 dpi. IL-10 can down regulate inflammatory Th responses via a regulatory CD4+ subset, Treg [27]. Both IL-10 and TGF-β are pleiotropic cytokines, which are generally regarded as anti-inflammatory. We observed synchronous up-regulation of IL-10 and TGF-β at 33 dpi (Fig. 2H). Possible differentiation of Th1 was indicated with changes of the transcript levels of IL-12β and beta-2-microglobulin-2 JB1 (B2M), a component of the major histocompatibility complex (MHC) class I (Fig. 2G, E). B2M was down-regulated 22 dpi and interestingly, similar changes were observed in skin of carp infected with ectoparasite *Ichthyophthirius multifiliis *[28]. Changes of CD8α suggested involvement of cytotoxic T cells (Fig. 2G). Arginase 1 (ARG1), a typical marker of alternatively activated macrophages (M2), central downstream effector cell of the Th2 response, implied the pronounced activation of M2 and their rapid recruitment both at the onset and at the end of infection (Fig. 2G). Activation of T cell-related genes 3 dpi was followed with decline 22 dpi and increase at 33 dpi in intact skin (Fig. 2F, G). Sarcoplasmic/endoplasmic reticulum calcium ATPase involved in calcium sequestration was up-regulated 22 dpi in injured areas and then down regulated 33 dpi (Fig. 1). A similar expression pattern was seen in several genes involved in calcium signaling and muscle contraction, including calcium-binding protein parvalbumin α2 and several myosin genes (Fig. 1).

Regulation of a number of genes with known anti-inflammatory actions was observed. Annexins were consistently up-regulated at the damaged sites (Fig. 1) [29]. Increase of prostaglandine D synthase (PGDS) expression 3 dpi was followed with down-regulation 33 dpi at the sites of SL attachment (Fig. 1 and Fig. 2E). PGDS is the key enzyme involved in the synthesis of PGD_2_, which is further metabolized to 15-d-PGJ_2_, a potent anti-inflammatory mediator [30]. It can inhibit the production of iNOS, TNF-α and IL-1β in macrophages through the inhibition of MAP kinases, nuclear factor kappaB (NFkB) or IkB kinase [31,32]. 15-d-PGJ2 mediates the inhibition of proliferative responses of T cells and induces apoptosis of T cells by a PPAR-γ-dependent mechanism [33].

Accumulation of misfolded proteins in endoplasmic reticulum lumen activates a set of intracellular signalling steps collectively called the unfolded protein response (UPR). UPR is induced by a variety of insults, including nutrient and oxygen deprivation, pathogen infections, changes in redox status and intralumenal calcium (reviewed in [34]). Microarray analyses showed highly significant induction of mitochondrial proteins involved in biosynthesis and transport of ATP, heat shock proteins, 94 kDa glucose-regulated protein (GRP94), 78 kDa glucose regulated protein (GRP78), and X-box binding protein 1 (XBP-1) indicating unfolded protein response (UPR), typical of wounded tissue (Fig. 1).

As expected, massive changes in genes for proteins of ECM took place and they were substantially greater in the damaged sites (Fig. 1 and Fig. 2A, B). In addition to MMPs, several lysosomal proteases, cathepsins were regulated, which was in line with the degradation of tissue (Fig. 1 and Fig. 2D). Secreted cathepsin S (CTSS) is an elastolytic cysteine protease capable of degrading ECM components. We observed down-regulation of several types of collagens and up-regulation of elastin and laminin in the damaged sites and these changes increased markedly at the late stage of SL development (Fig. 1 and Fig 2A, B). Gene expression changes that can be relevant to remodelling of ECM were not only observed in infected sites. Down-regulation of decorin (DCN) was found in intact skin 3 dpi and 22 dpi (Fig. 2B). Rapid changes were seen in the bone morphogenetic protein (BMP4), which was markedly suppressed until 33 dpi in both intact and injured sites (Fig. 2C). The enzymes involved in modification of extracellular matrix also showed rapid responses (Fig. 2D). Increase of alkaline phosphatase (ALP) activity was reported in mucus of SL infected Atlantic salmon [35] and in regenerated scales of a common goby [36]. In the present study, early decrease of ALP expression at the intact sites was followed with the up-regulation in all areas (Fig. 2C).

### Responses to salmon lice in the head kidney, liver and spleen

**Figure 3:**
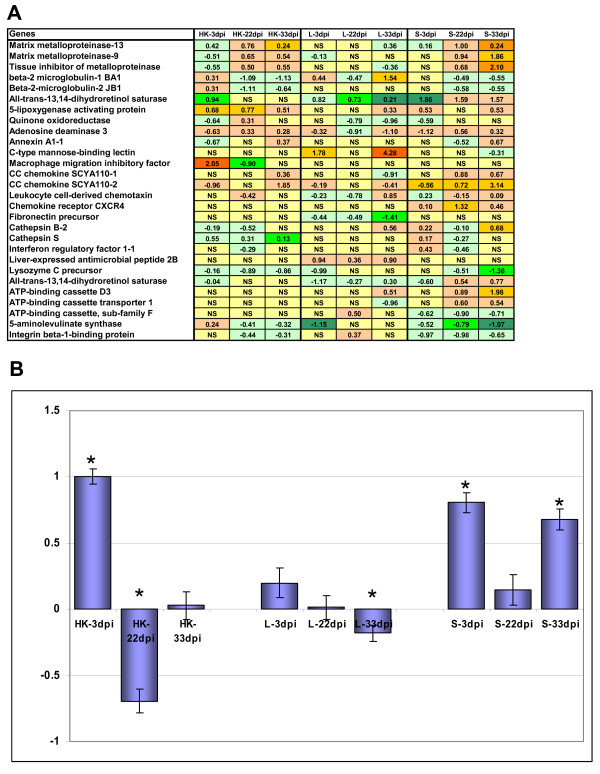
**Microarray analyses of gene expression in head kidney (HK), liver (L) and spleen (SP).** Pooled samples were analysed using uninfected fish as a common reference. **A **– log-expression ratios. Significantly up- and down-regulated genes (p < 0.05, t-test, 12 spot replicates per gene) are highlighted with red and green scales, NS is not significant. **B**: mean expression profiles of nine immunoglobulins designated by the Unigene clusters and most similar mammalian genes: Omy 9391 (Ig kappa chain V-III region VG), Omy 416 (Ig kappa chain V-IV region JI), Omy 23312 (Ig kappa chain V-IV region B17-1), Omy 9391 (Ig kappa chain C region), Omy 30091 (Ig kappa chain V-IV region Len), Ssa 709 (Ig kappa chain V-IV region B17-2), Ssa 78 (Ig heavy chain V-III region HIL), Omy 11287 (Ig mu heavy chain disease protein), Ssa 709 (Ig kappa chain V-I region WEA) Data are mean log-expression ratios ± SE, significant differences from zero (p < 0.05) are indicated with *.

**Figure 4:**
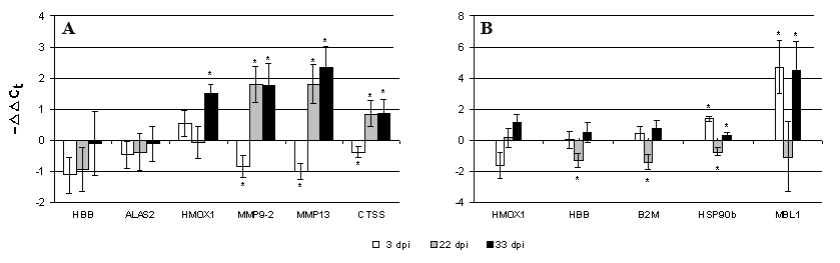
**Gene expression in spleen(A)and liver (B) analyzed with qPCR (individual samples). ** Data are -ΔΔCt ± SD (n = 6). Significant difference from uninfected control (t-test, p < 0.05) is indicated with *. **A**: spleen. Haemoglobin beta (HBB) and 5-aminolevulinate synthase (ALAS2) were selected for qPCR analyses as the representatives of genes involved in erythropoiesis and metabolism of iron. Two metalloproteinases (MMP) 9 and 13 engage in ECM remodelling and cathepsin S (CTSS) may indicate activation of the Th1-related adaptive immunity **B**: liver. Genes selected for qPCR analyses: heme oxygenase 1 (HMOX1) and HBB, involved in iron metabolism; beta-2-microglobulin-2 JB1 (B2M), an indicator of Th1 responses; heat shock protein 90 β (HSP90b), implied in cellular stresses; C-type mannose binding lectin (MBL1), receptor possibly involved in the recognition of SL-derived antigens. Significant difference from uninfected control (t-test, p < 0.05) is indicated with *.

The gene expression changes in the intact sites of skin suggested rapid systemic responses to parasites. This was supported by the results of analyses in the head kidney, spleen and liver (Fig. 3 and Fig. 4). These organs were included in the study due to their essential roles in immunity. Rapid change was seen in a group of immunoglobulin (IG) like genes, which then returned toward control levels in the head kidney (Fig. 3B). Early increase of the expression of these genes in the spleen declined during chalimus developmental stages, with subsequent return to a similar level to initial by day 33. Changes of MMPs (delayed increase in the head kidney and spleen first noticed at 22 dpi) were one of the most remarkable features of the infected fish (Fig. 3A and Fig. 4A). In our earlier studies, similar MMP profiles were observed in rainbow trout challenged with handling stress [14]. Differential expression of B2M was seen in all studied tissues of SL-infected fish and only in the liver these genes were up-regulated (Fig. 3A). Quinone oxidoreductase and all-trans-13,14-dihydroretinol saturase were progressively down-regulated in the liver while opposite was seen for quinone oxidoreductase in the head kidney and for all-trans-13,14-dihydroretinol saturase in the spleen (Fig 3A). Adenosine deaminase is a regulator of inflammation [37,38], which was down-regulated in liver 22 dpi, while it had a peak in expression at the same stage of infection in the head kidney and spleen (Fig. 3A). C-type mannose binding lectin (MBL1) was up-regulated in liver but only 3 dpi and 33 dpi (Fig. 3A and Fig. 4B). MBL recognises carbohydrates on both foreign organisms and damaged cells and cellular debris and then initiates their removal and local inflammatory responses. The 5-lipoxygenase activating protein was markedly activated in head kidney at all stages but only 33 dpi in spleen (Fig. 3A). This protein is required for the production of leukotrienes, best known for their potent chemotactic and leukocyte-activating effects [39,40]. CXC chemokine receptor (CXCR4) has a potent chemotactic activity for lymphocytes and was shown to inhibit haematopoietic stem cell proliferation [41]. In the present study, up-regulation of CXCR4 in spleen 22 dpi was observed (Fig. 4A). A suit of other chemokines with potential roles in recruitment and activation were regulated in internal organs (CC chemokine SCYA110-1, CC chemokine SCYA110-2, leukocyte cell-derived chemotaxin 2 and macrophage migration inhibitory factor-like) (Fig. 3A).

Co-ordinated changes were seen in the genes involved in metabolism of iron and erythropoiesis. In head kidney and spleen this group was down-regulated within whole study period while in liver, initial decrease was followed with the gradual elevation. Selected representatives for qPCR analyses in spleen were haemoglobin beta chain (HBB) and erythroid-specific 5-aminolevulinate synthase (ALAS2) (Fig. 4A) and heme oxygenase 1 (HMOX1) and HBB in liver (Fig. 4B).

## Discussion

Results of this study represent a significant contribution into the understanding of the underlying physiological basis for the high susceptibility of Atlantic salmon to salmon lice and the side effects caused by the infection with this parasite. The ability to reject parasites can be determined with inflammation and healing of wounds, and here, we observed expression changes of genes involved in these processes.

### ECM and wound healing

The pathology of Atlantic salmon infected by high numbers of lice is characterised by gross lesions, vast areas of eroded skin on the head and back, necrotic tissue and sub-epidermal haemorrhaging at margins of lesions (reviewed in [1]). Because of the danger of osmotic shock in aqueous environment, any break in the fish skin must be rapidly repaired. The initial hemostatic event upon breaching of epidermis provides the provisional fibrin-fibronectin wound matrix, which is a framework for cell adhesion, migration, and repair [42]. Maintaining sufficiently high levels of plasma fibronectin, produced in the liver, plays an important role in wound healing [43]. Fibronectin was down-regulated in liver of Atlantic salmon already 3 dpi and continued until 33 dpi, suggesting limited wound healing ability in the afflicted skin (Fig. 3A). Fibronectin is a large adhesive glycoprotein which interacts with cells and transmits signals primarily through integrin receptors expressed on a variety of epidermal cells including keratinocytes, endothelial cells and fibroblasts, allowing them to interweave with the fibrin clot in the wound space [44,45]. In a fish scale-skin culture system, dermal substrates such as fibronectin and type I collagen were able to initiate migration of keratinocytes and epidermal outgrowth even in the absence of growth factors [46]. In the present study, expression profiles of genes encoding ECM components were also similar to profiles characteristic for slowly repairing injuries [47-49]. This included down-regulation of several collagens at the end of experiment. A slight but consistent up-regulation of COL1a was detected by qPCR, however, COL10a was stably down-regulated as well as COL2a at 3 dpi and 22 dpi. A relatively low induction of decorin, a regulator of assembly of collagen fibrils [50,51] and TGF-β activity [52,53], was seen 22 dpi and similar changes were observed in ALP. However marked up-regulation of major ECM proteins elastin and laminin was seen only 33 dpi as well as induction of COL2a. Noteworthy, slow reparation of ECM was in parallel with stable up-regulation of MMP9 and MMP13 in the damaged sites, whose excessive activity may contribute to the development of chronic wounds [54].

Delayed wound healing could be accounted for by the insufficient expression of several regulatory proteins. Increase of TGF-β, an essential stimulator required for ECM development [55], was seen not earlier than 33 dpi. Actions of TGF-β, which is released from platelets and macrophages immediately after injury, largely depend on the presence of fibronectin and other ECM components [53] and vice versa. It attracts neutrophils, macrophages, and fibroblasts, which in turn release more TGF-β. Relatively modest changes in matrix composition were shown to have major effects on cell responses and growth [56], including self-renewal [57]. We studied the expression of a developmentally important gene, BMP4, another TGF superfamily member, which is known to be up-regulated in undifferentiated stem-like cells [58] and to play an important role in skin homeostasis [59]. This gene was markedly down-regulated until 22 dpi. Collectively, these gene expression profiles of skin ECM and MMPs classify the wounds afflicted by SL as chronic, due to the significantly protracted duration and deregulation of events in the healing cascade. Together with opposite expression changes of several genes in the intact and damaged sites (MMPs, C/EBPb) this could indicate a modulatory activity of SL and/or constant damage inflicted by the growing parasites. The ability of SL to reduce the protective responses has been reported by several groups. Firth et al. (2000) [60] characterised low molecular proteases (LMW) secreted by *L. salmonis *onto the surface of the fish as trypsins, which are known to be used by many parasites for invasion and to suppress immune responses in their hosts [61]. Fast et al. [11] correlated the reduced respiratory burst and phagocytosis in macrophages of infected Atlantic salmon and rainbow trout with the appearance of LMW bands in the mucus.

### Inflammatory responses

The rate of wound healing and the ability to reject parasites could also be explained by the characteristics of inflammatory responses to SL. Inflammation is regarded as a two-edged sword since destructive alterations are closely associated with the subsequent reparation of damaged tissues and often involve the same or closely related molecular mechanisms and cellular elements. Hence, suppression of inflammation may result in chronicity. In SL infected coho salmon, tissues develop pronounced epidermal hyperplasia at the sites of attachment, which is accompanied with abundant cellular infiltration within the dermis beneath chalimii [1,8]. Inflammatory infiltrate consists mostly of neutrophils. Cellular debris and phagocyte neutrophils are abound at the early phases of infection whereas macrophages and a small number of lymphocytes appear later. Collectively, the findings in these studies are in line with our results showing restricted tissue inflammatory changes at both copepodid and chalimus stages. Early up-regulation of immunoglobulin-like genes in the head kidney and spleen, in addition to a panel of immune genes in the intact areas of skin, indicated a rapid activation of the systemic antiparasitic responses. In SL infected skin, up-regulation of Sp1 cofactor, a partner of NFkB [62], provided indirect evidence for possible activation of the NFkB pathway. An inflammatory state could also explain the decreased expression of quinone oxidoreductase in SL skin 33 dpi, as was similarly observed in mammalian cells [63]. However, the input of NFkB pathway in responses to SL was relativley low. At low level of NFkB activation, T cells develop an anergic state through Ca2+ signalling [64]. In this respect, it is noteworthy to mention that SL infection induced a number of Ca2+ regulatory genes, e.g. calcineurin, calmodulin and calpactain I light chain (Fig. 1B).

Restricted inflammatory responses in SL damaged skin were in parallel with massive up-regulation of chaperones indicative of the induction of UPR. In addition, increased levels of genes regulating mitochondrial proteins were observed. This has been previously observed in relation to responses to handling stress [14] and acute toxicity [13,65] in rainbow trout. Such opposite regulation of stress and immune responses is well known. Cortisol, the most common marker of stress in fish is widely used for anti-inflammatory therapy. Increased levels of cortisol that were reported in lice infected salmon [12,35,66] can at least partly account for the lack of strong inflammation. Steady increase of MMPs in all organs except liver was a remarkable feature of SL infected salmon. In salmonid fish these genes are up-regulated with both stress and inflammatory agents [14,67]. Prolonged stress, infection and combination of these two can result in chronic degradation of ECM.

### What immune cells can be involved in responses to SL?

Microarray analyses revealed signs of inflammation but did not indicate which immune cells could have taken part in responses to SL. To address this, a set of markers was included in the qPCR analyses. ARG1, a marker of alternatively activated macrophages (M2) [68] with an important role in tissue remodelling and wound healing [69], was up-regulated 3 and 33 dpi but down-regulated 22 dpi at the intact sites. In contrast to classically activated macrophages (M1), M2 are induced by Th2 cytokines IL-4 and IL-13, that are prevalent at parasitic infections and in wound settings [70,71]. The maintenance of M2 requires the adaptive arm of the immune system [72]. Therefore, decreased ARG1 expression 22 dpi suggested the possibility of insufficient signaling from the responsible T cell subset (Th2) at this stage.

Responses to parasites are often described in terms of Th1/Th2 dichotomy but recent studies have shown that host-pathogen interactions are more complex. A novel T cell effector subset Th17, characterised by the production of IL-17 was identified as well as a regulatory T cell subset (Treg), its signature cytokines being inhibitory IL-10 and/or TGF-β [73]. In mammals Th1, Th2 and Th17 reciprocally regulate the development and function of each other, while Treg cells suppress all three subsets [74,75]. The regulatory cytokines control inflammation and thus protect against immunopathology, but, in so doing, they reduce the effectiveness of immune mechanisms responsible for the expulsion of the parasites. Th2-dependent immune effector mechanisms are diverse and include fibroblast recruitment to the damaged tissue and collagen production, smooth muscle hypercontractivity and accelerated epithelial cell turnover [70,71,76]. Numerous studies of fish-parasite interactions described physiological responses, typically induced in a Th2 setting, e.g. increased mucus production and goblet cell hyperplasia in *Gyrodactilus *and SL infection models [1,5,77].

**Figure 5:**
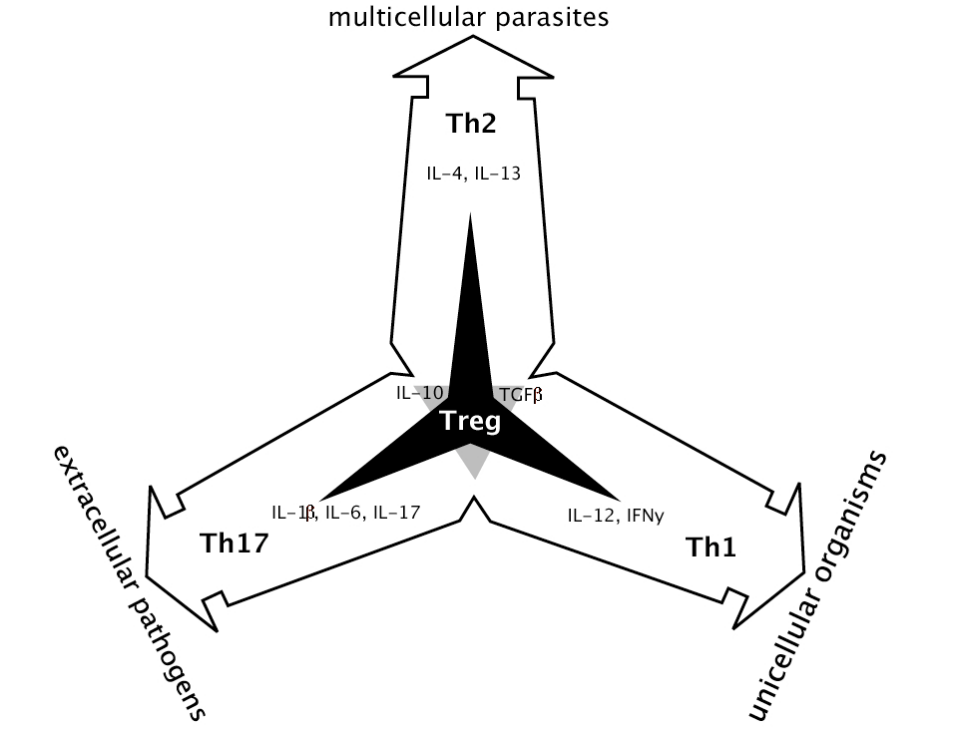
**A map of T cell-mediated responses to pathogens.** Three effector subsets, Th1, Th2, Th17, and the regulatory Treg are characterized by distinct cytokine profiles [73]. All three pro-inflammatory subsets reciprocally antagonize each other as indicated with a grey triangle in the centre where they were shown to overlap. Treg cells, represented with a black three-pointed star superimposed at the centre of the figure, inhibit all three Th subsets, thus preventing excessive inflammatory responses. T cell-mediated responses represent combined immune responses, which include both innate and adaptive components. Immune response to most bacterial and viral pathogens is generally pro-inflammatory. The Th1 cells secrete interferon γ (IFNγ) and IL-12, which protect against viral infections and other intracellular pathogens. Th17 is a highly pro-inflammatory arm characterised by rapid induction of neutrophils at the inflamed tissue and requires IL-1 and IL-6 for its activation. In contrast, parasitic infections drive Th2 immune responses characterized by production of IL-4 and IL-13, which mediate elimination of multicellular parasites. In addition to driving polarized Th2 responses, parasitic infections are associated with the induction of Tregs and immunoregulatory IL-10, which can induce immune anergy. The resultant effect of this is that parasitic infections can be characterized by an overall down-regulated immune system and therefore modified Th2-response, termed Th2-like. Because many cytokines can be produced and utilised by a number of different cells (IL-10 being a good example [106]), it is clear that multiple cell types may contribute to the regulation of the type and extent of inflammation. Thus, immune regulation likely depends on the specific combination of different T cells called upon during an infection than on a clear predominance of one response profile.

It is becoming increasingly evident that a superimposition of polarized response profiles in parasitic infections is the norm rather than the exception [75,78]. Concurrent regulation of markers of opposing immune responses in this study adds to this notion, e.g. early T cell response occurred in both the CD4+ and CD8+ T cell compartment (Fig. 2F, G). We visualized the map of immune responses which can accommodate for any particular combination of activated T cell responses (Fig. 5). In this map, each of the four major T cell-mediated responses can overlap with any other one and more than a combination of two is possible. Although Th1, Th2, Th17 and Treg are regulated with the lineage-specific sets of cytokines and respond to different targets, they can coexist in combinations. Th1 and Th2 responses can overlap with anti-inflammatory Treg or, quite the opposite, with the Th17 responses.

A specific set of innate effector cells is summoned by each of these T cell subsets. The newly described Th17 developmental pathway is characterised by the rapid neutrophilia [79]. The Th17 is thought to be an ancient lineage highly conserved in all vertebrates including the jawless lamprey [80,81]. This highly pro-inflammatory CD4+ Th subset plays an important role in the immediate responses to injuries with high risk of necrosis [82], and in protection against extracellular pathogens which are not efficiently cleared by Th1-type and Th2-type immunity [83]. The outcome of parasitic infections may be determined by the balance of pro-inflammatory and regulatory immune responses. Our observations indicate that reactions to SL in Atlantic salmon are consistent with the bias towards the regulatory/Th2-like responses.

Identification of genes involved in the Th1/Th2 axis was greatly enhanced with the sequencing of several fish genomes [80,84]. The cytokine networks are becoming increasingly better known in fish. Overall, fish possess a repertoire of cytokines, which is similar to mammalian [85,86], including recently cloned IL-6 [87,88], IL-12 [89] and IL-10 [90-93]. First Th2 type interleukin, IL-4 was cloned in 2007 [94]. Also recently, the master regulators of Th1 and Th2 development, T-bet and GATA-3, respectively, were described in fish [95,96]. Cytokine profiles alone are insufficient for the accurate assignment of T cells to lineages given that cytokines may work in different ways. For example TGF-β induces development of both Treg and Th17 in mice and suppresses Treg development in humans [97]. One may anticipate much greater differences between mammals and teleost fish.

Rapid involvement of T cells in response to infection in skin was implied already 3 dpi. However, the delayed healing in SL infected Atlantic salmon may indicate an impaired or modified, Th2-like response. Long lived parasites often cause chronic infections via the induction of Treg cells and concomitant down-regulation of protective Th responses [71]. The involvement of a Treg-like subset, as implied by the up-regulation of TGF-β and IL-10 at 33 dpi, was not seen in the chronic phase of SL infection. This coincided with the molting of pre-adults into mobile adult stages. Though probably down modulatory towards the effector Th arms in fish, IL-10 and TGF-β may benefit the host at this stage due to the reduced damage caused by inflammatory reactions and/or enhanced healing response. The augmented healing would not harm SL either, as most chalimii molted into mobile life stages 33 dpi and maintenance of an open wound to aid in feeding may not have been necessary. We also observed the regulations of gene expression in the internal organs, which were consistent with changes in the skin. Early activation of the humoral response, as evidenced by the transcriptional wave of immunoglobulin-like genes, was followed by general hyporesponsiveness during immobile SL stages 22 dpi (Fig. 3B and 3A). Expression profiles of immunoglobulins partially reverted 33 dpi only in the spleen.

**Figure 6:**
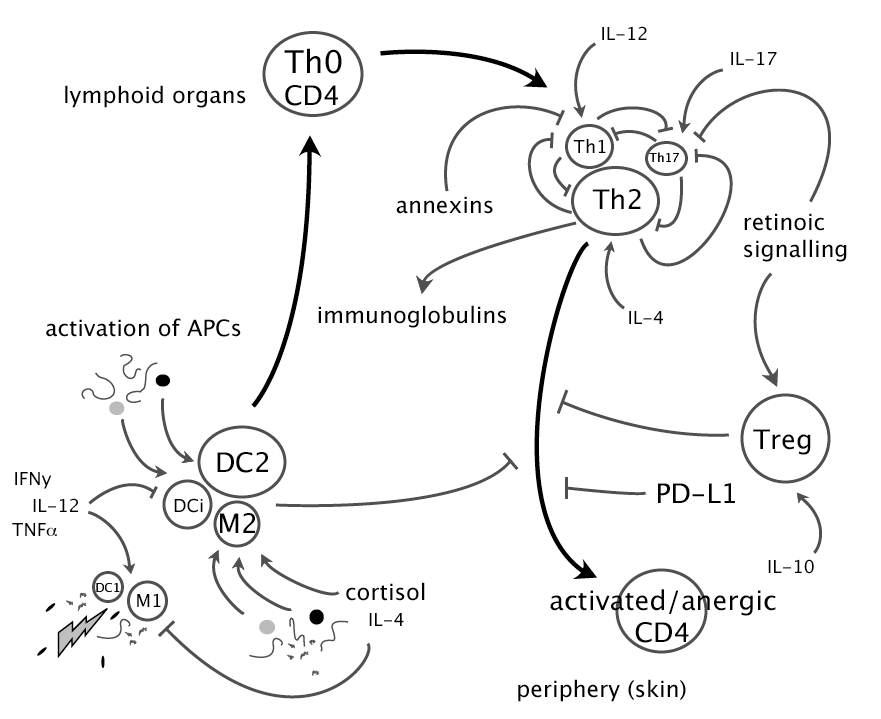
**Hypothetical model of responses of immune cells to SL.** Classical activation of macrophages (M) and dendritic cells (DC) induces M1 and DC1 phenotypes, which drive CD4+ T cells toward Th1. In contrast, SL may selectively induce C-type lectin receptors and possibly other receptor classes on alternatively activated macrophages (M2), immature DC (DCi) and DC2 to preferentially induce Th2 cells [75]. Parasite antigens are presented to CD4+ T cells in lymphoid tissues. Upon activation, Th2 cells proliferate and induce immunoglobulin production, possibly down-regulating other Th subsets. However, different subsets can coexist and a balanced combination may result in susceptibility or resistance. Activated Th2 cells migrate to the site of SL attachment where they mediate expulsion of chalimus larvae. Antagonism within the Th compartment and suppression by Treg cells can inhibit CD4+ T cell effector functions. Several other mechanisms can have key functions in the shaping of the T cell repertoire, and in regulation of inflammatory responses to SL, including reciprocal Th17 and Treg differentiation mediated by vitamin A derivatives [107] and various anti-inflammatory agents, such as annexins. Programmed death ligand 1 (PD-L1) possibly provides a distinct negative regulatory checkpoint in T cell differentiation [98]. Endogenous products (e.g. cortisol and prostaglandins), cellular debris and SL products are also able to potently influence immune responses [108-110].

Possible roles of cells and the regulatory network are presented in Figure 6. Interestingly, the expression profile of programmed death ligand 1 (PD-L1), a negative co-stimulatory signal of T cell activation [98], was similar to that of CD4. The differential regulation of all-trans-13,14-dihydroretinol saturase in skin, liver and spleen also implies a dynamic regulation of immune cells through retinol signalling (Fig. 1B and Fig. 3A). Vitamin-A deficiency is known to induce immune abnormalities in T-cell subsets [99-101]. Retinoic acid signalling was recently shown to inhibit Th17 and promote Treg differentiation [102]. Interestingly, two structurally novel protein families with a high affinity to retinol and fatty acids, potentially playing a role in modifying inflammatory environment were identified in parasitic nematodes [103,104].

The skin expression profile of cytochrome P450 27, which ties together retinoid, PPARγ and LXR signaling [105] implies the down regulation of a whole regulatory network based on natural/endogenous ligands: retinoids and modified fatty acids and prostanoids (Fig. 1B). Of note is the observation that all-trans-13,14-dihydroretinol saturase is down regulated in skin and liver but from 22 dpi becomes up-regulated in spleen. In addition, 5-lipoxygenase binding protein, involved in the generation of leukotrienes, another set of lipid mediators of immunity was up-regulated throughout the study period in head kidney. Such expression profiles may stem from the fact that skin is under the direct and stronger modulation by the parasite than the internal organs.

**Figure 7:**
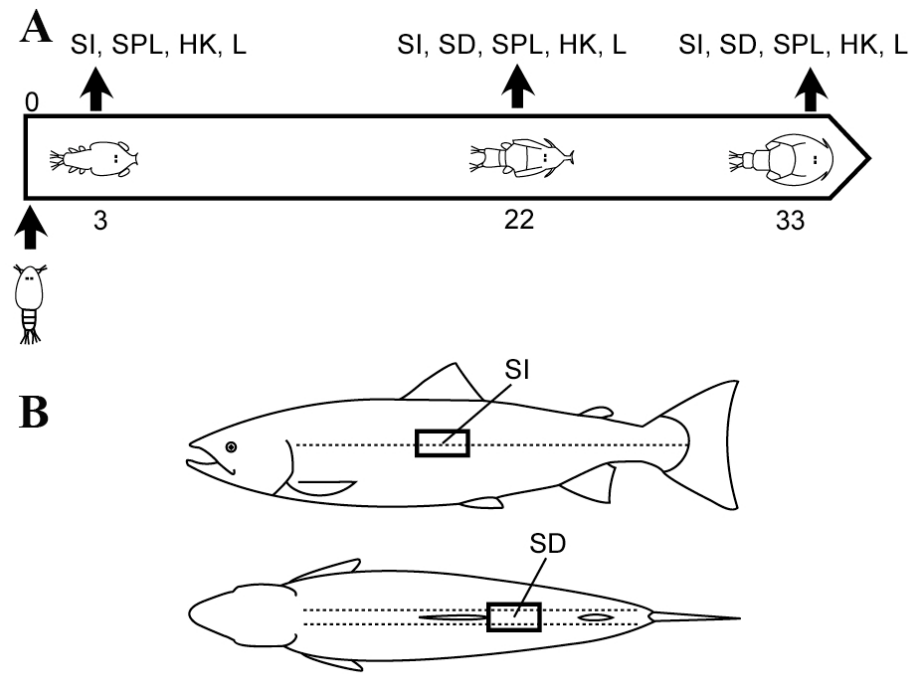
**Design of the experiment.A**: Two study groups of Atlantic salmon were SL infected fish with approximately 75 copepodids per fish. On days 3, 22 and 33 tissue samples were collected from the control and infected test groups; time-points corresponded to copepodids (3 dpi), chalimus III/IV (22 dpi) and preadult stages (33 dpi). **B**: Due to the small size of SL at the copepodid stage, it was not possible to reliably locate them on fish at the sample taken 3 dpi. Consequently, SL damaged skin was only sampled 22 dpi and 33 dpi. Salmon lice tend to aggregate around fins. Therefore, the area behind the dorsal fin was chosen in order to sample damaged skin. Because attached lice were never found along the lateral line, samples of intact skin of infected fish were taken from the area below the dorsal fin at the intersection with the lateral line. SI – intact skin; SD – damaged skin; SPL – spleen; HK – head kidney; L – liver; dpi – days post infection.

## Conclusion

Initial infection of Atlantic salmon with SL is associated with rapid sensing and induction of mixed inflammatory responses. A combination of restricted inflammation, which can be due to hyporesponsiveness of the immune cells, and delayed healing of injuries, can account for the limited ability to reject parasites. Persistent infection of Atlantic salmon with SL implies compromised immunity and self-destruction of tissues. Development of markers for different subsets of salmon T cells will greatly enhance the opportunity to study responses of Atlantic salmon to SL, and other parasites.

## Materials and methods

### Challenge test

Salmon used for this experiment originated from the Aqua Gen AS (previously referred to as NLA) strain. Salmon smolts of mixed and unknown family background were transferred to Institute of Marine Research in Bergen, transferred to full salinity saltwater, and distributed into two replicate tanks (two control and two for *L. salmonis *infection). Fish were hand fed a commercial diet once daily during the entire experimental period. Water temperature was 10°C ± 1.5°C during the entire experimental period. Egg strings from hatchery reared SL were collected and placed into single incubators. Approximately 75 infectious copepodids/fish were used to infect 40 salmon (20/tank). On the day of infection, the water level in the replicate tanks was reduced to approximately one third of the tanks original volume, the water supply was stopped, and the water was aerated with oxygen. One hour post infection, the water supply was reinstated and oxygen supply to the tanks removed.

The experimental fish were sampled on the following dates: 21.09.2006 (3 dpi = copepodids), 11.10. 2006 (22 dpi = chalimus III/IV), and 21.10.2006. (33 dpi = pre-adult females and males) (Fig. 7A). At 22 dpi the number of lice per fish ranged from 4 to 20 with mean 12.2 ± 1.8. Individuals with average numbers of parasites were used for analyses. Control fish were sampled in parallel with challenged fish. In addition to skin, samples of the head kidney, spleen and liver were collected and preserved in RNALater (Ambion, Austin, TX, USA) (Fig 7B). Lice induced damage to the fish was moderate, and no open wounds were observed on any of the experimental fish. Furthermore, there was no evidence of any secondary infections either on the surface or internal organs for the infected or control fish.

### Microarray analyses

The salmonid fish microarray (SFA2, immunochip) includes 1800 unique clones printed each in six spot replicates. The genes were selected by their functional roles and the platform is enriched in a number of functional classes, such as immune response (236 genes), cell communication (291 genes), signal transduction (245 genes), protein catabolism (90 genes) and folding (70 genes). The complete composition of platform and sequences of genes are provided in submission to NCBI GEO (GPL6154). Total RNA was extracted from soft tissues with TriZOL (Invitrogen, Carlsbad, CA, USA), whilst Fibrous tissue kit (Qiagen sciences, Maryland, USA) was used with skin. Total RNA was purified with Pure Link (Invitrogen, Carlsbad, CA, USA). Microarray analyses were conduced in pooled samples with equal inputs of RNA from six individuals. A dye swap design of hybridization was applied. In analyses of injured skin, the intact sites from the same individuals were used as a control. Microarray comparisons were also conducted with intact skin from challenged and control fish; these data were not reported due to relatively small expression changes. Analyses of head kidney, spleen and liver used the uninfected fish as a reference. Each sample was analyzed with two slides. The control and test samples (20 μg RNA in each) were labelled with respectively Cy3-dUTP and Cy5-dUTP (Amersham Pharmacia, Little Chalfont, UK) for the first slide and dye assignment was reversed for the second slide. The fluorescent dyes were incorporated in cDNA using the SuperScript™ Indirect cDNA Labelling System (Invitrogen, Carlsbad, CA, USA). The cDNA synthesis was performed at 46°C for 3 hours in a 20 μl reaction volume, following RNA degradation with 0.2 M NaOH at 37°C for 15 min and alkaline neutralization with 0.6 M Hepes. Labelled cDNA was purified with Microcon YM30 (Millipore, Bedford, MA, USA). The slides were pretreated with 1% BSA fraction V, 5× SSC, 0.1% SDS (30 min at 50°C) and washed with 2 × SSC (3 min) and 0.2 × SSC (3 min) and hybridized overnight at 60°C in a cocktail containing 1.3 × Denhardt's, 3 × SSC 0.3% SDS, 0.67 μg/μl polyadenylate and 1.4 μg/μl yeast tRNA. After hybridization slides were washed at room temperature in 0.5 × SSC and 0.1% SDS (15 min), 0.5 × SSC and 0.01% SDS (15 min), and twice in 0.06 × SSC (2 and 1 min, respectively). Scanning was performed with GSI Lumonics ScanArray 4000 (PerkinElmer Life Sciences, Zaventem, Belgium) and images were processed with GenePix 6.0 (Axon, Union City, CA, USA). The spots were filtered by criterion (*I-B*)/(*S*_*I*_*+S*_*B*_) ≥ *0.6*, where *I *and *B *are the mean signal and background intensities and *S*_*I*_, *S*_*B *_are the standard deviations. Low quality spots were excluded from analysis and genes presented with less than three high quality spots on a slide were discarded. After subtraction of median background from median signal intensities, the expression ratios (ER) were calculated. Lowess normalization was performed first for the whole slide and next for twelve rows and four columns per slide. The differential expression was assessed by difference of the mean log-ER between the slides with reverse labelling (6 spot replicates per gene on each slide, Student's t-test, p < 0.01). Complete microarray results are provided as an additional file 1.

### Quantitative real-time RT-PCR

The cDNA synthesis was performed on 2 μg of DNAse treated (Turbo DNA-*free*™ (Ambion, Austin, TX, USA) total RNA using TaqMan^® ^Reverse Transcription reagents (Applied Biosystems, Foster City, CA, USA) and random hexamer primers. The PCR primers (Table 1) were designed using the Vector NTI (Invitrogen) and synthesized by Invitrogen. PCR. Efficiency was checked from tenfold serial dilutions of cDNA for each primer pair (additional file 2). Real-time PCR assays were conduced using 2X SYBR^® ^Green Master Mix (Roche Diagnostics, Mannheim, Germany) in a 12 μl reaction volume, primer concentrations were 0.4–0.6 μM each. PCR was performed in duplicates in 96-well optical plates on Light Cycler 480 (Roche Diagnostics). Relative expression of mRNA was calculated using the ΔΔC_T _method; the chosen reference gene for all tissues was eukaryotic translation initiation factor 3 subunit 6 (eIF3S6), which showed no differential expression according to the microarray results. Two more commonly used genes (EF1A and GAPDH) were tested for stability using the GeNorm software, however only eIF3S6 met criteria of stability in the analyzed material. Differences between infected and control fish were analyzed with Student's t-test (p < 0.05).

**Table 1:** Real-time qPCR analyses

**Target**	**Primer sequence from 5' to 3'**	**Amplicon size (bp)**	**Accesion number**
Matrix metalloproteinase 13	F CCAAAAAGAGGGCACCAGATGG	53	DW539943
	R CCAAAAAGAGGGCACCAGATGG		
Matrix metalloproteinase 9	F AGTCTACGGTAGCAGCAATGAAGGC	72	CA342769
	R CGTCAAAGGTCTGGTAGGAGCGTAT		
Cathepsin S	F CGAAGGGAGGTCTGGGAGAGGAAT	87	CA355014
	R GCCCAGGTCATAGGTGTGCATGTC		
Bone morphogenetic protein 4	F TCAAGTTGCCCATAGTCAGT	207	CA056395
	R CACCTGAACTCTACCAACCA		
Alkaline phosphatase	F CTAGTTTGGGTCGTGGTATGT	185	CA358635
	R TGAGGGCATTCTTCAAAGTA		
Heat shock protein 90β-20	F GAACCTCTGCAAGCTCATGAAGGA	72	CF752846
	R ACCAGCCTGTTTGACACAGTCACCT		
Collagen 10α	F TGGTGCTCTTTGACTGCCTGTAA	180	EG837148
	R CATCCTGTGTGTTGCAATATCACA		
Collagen 1α	F AGAGAGGAGTCATGGGACCCGTT	155	
	R GGGTCCTGGAAGTCCCTGGAAT		
Collagen 2α	F TGGTCGTTCTGGAGAGACT	151	BX865386
	R CCTCATGTACCTCAAGGGAT		
Decorin	F GAACCTGGCTAAGCTGGGTCTAA	256	DQ452069
	R GAACAGGCTGATGCCAGAGTACAT		
Elastin	F GAGGCTACAGACCAGGAGGAGTT	226	BU694149
	R TCTGGGTCGGTGGGTTTGTA		
Laminin	F CATGTGACATGGACACAGGAA	273	DY722974
	R CGTCCTCAGCCTCATAGGTGTA		
CCAAT/enhancer binding protein β	F TACGTCCTGGGCTATCCTGAACTGC	140	CA348284
	R CCAGACGAACCGTTGTTGTCCA		
Erythroid 5-aminolevulinate synthas	F CACATGAGACAGCTGCTCCTGGAGA	121	DW580939
	R GCTCCAGCAAGATGTCACACACCT		
Heme oxygenase 1	F AGTCAGTGGAGAGAGACCTGGAGCA	117	CA363120
	R GGTTGTCTTTGCCGATCTGTCTGAG		
Haemoglobin beta chain	F ACAAACGTCAACATGGTCGACTGG	67	NM_001123666
	R TCTTTCCCCACAGGCCTACGAT		
Mannose binding lectin 1	F TCCATTGCACTGGGCGATGC	105	CA349943
	R CACTGCTTCCACCTGAGCCTCA		
Prostaglandin D synthase	F CCTACACCAACCTGAACGCTGATG	98	CA352578
	R ACGCTGGCTGGTGAAGGTGAAG		
MHC class II α chain	F AGTCAGGTGGACCAGGAACAATCA	96	CA379977
	R CTGGAGAACTGGTTGAGGGTGAAA		
CD8α	F CGTCTACAGCTGTGCATCAATCAA	266	AY693391
	R GGCTGTGGTCATTGGTGTAGTC		
IL-12β	F TCTACCTACACGACATTGTCCAGCC	62	AJ548830
	R ATCCATCACCTGGCACTTCATCC		
Arginase 1	F AGCCATGCGTATCAGCCAA	122	EG929369
	AAGGCGATCCACCTCAGTCA		
Programmed death ligand 1	F TCAACGACTCTGGGGTGTACCGATG	133	CA366631
	R TCCACCTCATCTCCACCACGTCTC		
Beta-2-microglobulin	F TCGTTGTACTTGTGCTCATTTACAGC	107	AF180478
	R CAGGGTATTCTTATCTCCAAAGTTGC		
TGF-β	F AATCGGAGAGTTGCTGTGTGCGA	332	EU082211+
	R GGGTTGTGGTGCTTATACAGAGCCA		AJ007836
IL-1 receptor type 1	F CCAAAAAGAGGGCACCAGATGG	126	NM_001123633
	R CGTATCGTCTCTCCAACACCTCAGG		
CD4	F TGCATTGTTCCTCTCTTCCACAGC	128	EG852912
	R CCGTCCCAAGGTACCATAGTACCAA		
IL-10	F ATGAGGCTAATGACGAGCTGGAGA	54	EF165028
	R GGTGTAGAATGCCTTCGTCCAACA		
Eukaryotic translation initiation factor 3 subunit 6	F GTCGCCGTACCAGCAGGTGATT	92	CX040383
	R CGTGGGCCATCTTCTTCTCGA		

## Competing interests

The authors declare that they have no competing interests.

## Authors' contributions

All authors contributed to the overall experimental design. KG and FN designed and performed the challenge tests, whilst SS and AK carried out the gene expression analyses, data analysis, and produced the first manuscript draft. All authors read, contributed to, and approved the final manuscript.

## Supplementary Material

Additional file 1 **Complete results of microarray analyses.** Data are log (Expression ratios) and p-values of differential expression (t test). Click here for file

Additional file 2 **Efficiency of PCR.** Click here for file
