# Supplementary material for: Local and systemic gene expression responses of Atlantic salmon (Salmo salar L.) to infection with the salmon louse (Lepeophtheirus salmonis)
Source: BMC Genomics. 2008 Oct 23;9:498. doi: 10.1186/1471-2164-9-498 (PMC2582245; doi:10.1186/1471-2164-9-498)
Supplement: Additional file 2 — Efficiency of PCR. [file 1471-2164-9-498-S2.doc]

| **Target** | **PCR efficiency** | | |
| --- | --- | --- | --- |
|  | **skin** | **spleen** | **liver** |
| Matrix metalloproteinase 13 | 1.838 | 1.732 |  |
| Matrix metalloproteinase 9 | 1.785 | 1.690 |  |
| Cathepsin S | 1.710 | 1.814 |  |
| Bone morphogenetic protein 4 | 1.666 |  |  |
| Alkaline phosphatase | 1.613 |  |  |
| Heat shock protein 90β-2 |  |  | 1.782 |
| Collagen 10α | 1.674 |  |  |
| Collagen 1α | 1.734 |  |  |
| Collagen 2α | 1.817 |  |  |
| Decorin | 1.831 |  |  |
| Elastin | 1.703 |  |  |
| Laminin | 1.733 |  |  |
| CCAAT/enhancer binding protein β | 1.748 |  |  |
| Erythroid 5-aminolevulinate synthase | 1.775 | 1.699 |  |
| Heme oxygenase 1 |  | 1.722 | 1.881 |
| Haemoglobin beta chain |  | 1.668 | 1.767 |
| Mannose binding lectin 1 |  |  | 1.786 |
| Prostaglandin D synthase | 1.735 |  |  |
| MHC class II α chain | 1.670 |  |  |
| CD8α |  |  |  |
| IL-12β | 1.646 |  |  |
| Arginase 1 | 1.786 |  |  |
| Programmed death ligand 1 | 1.660 |  |  |
| Beta-2-microglobulin | 1.608 | 1.620 | 1.774 |
| TGF-β | 1.714 |  |  |
| IL-1 receptor type 1 | 1.679 |  |  |
| CD4 | 1.911 |  |  |
| IL-10 | 1.703 |  |  |
| Eukaryotic translation initiation factor 3 subunit 6 | 1.799 | 1.678 | 1.681 |
